# The Genealogic Tree of Mycobacteria Reveals a Long-Standing Sympatric Life into Free-Living Protozoa

**DOI:** 10.1371/journal.pone.0034754

**Published:** 2012-04-12

**Authors:** Otmane Lamrabet, Vicky Merhej, Pierre Pontarotti, Didier Raoult, Michel Drancourt

**Affiliations:** 1 URMITE CNRS-IRD UMR 6236, IFR48, Méditerranée Infection, Aix-Marseille Université, Marseille, France; 2 Equipe Evolution Biologique et Modélisation UMR 6632, IRF48, Aix-Marseille Université/CNRS, Marseille, France; University of Padova, Italy

## Abstract

Free-living protozoa allow horizontal gene transfer with and between the microorganisms that they host. They host mycobacteria for which the sources of transferred genes remain unknown. Using BLASTp, we searched within the genomes of 15 mycobacteria for homologous genes with 34 amoeba-resistant bacteria and the free-living protozoa *Dictyostelium discoideum*. Subsequent phylogenetic analysis of these sequences revealed that eight mycobacterial open-reading frames (ORFs) were probably acquired via horizontal transfer from beta- and gamma-Proteobacteria and from Firmicutes, but the transfer histories could not be reliably established in details. One further ORF encoding a pyridine nucleotide disulfide oxidoreductase (pyr-redox) placed non-tuberculous mycobacteria in a clade with *Legionella* spp., *Francisella* spp., *Coxiella burnetii*, the ciliate *Tetrahymena thermophila* and *D. discoideum* with a high reliability. Co-culturing *Mycobacterium avium* and *Legionella pneumophila* with the amoeba *Acanthamoeba polyphaga* demonstrated that these two bacteria could live together in amoebae for five days, indicating the biological relevance of intra-amoebal transfer of the pyr-redox gene. In conclusion, the results of this study support the hypothesis that protists can serve as a source and a place for gene transfer in mycobacteria.

## Introduction

Massive sequencing revealed that bacterial genomes have undergone a mosaic evolution, combining variable proportions of vertically acquired DNA from previous generations and horizontally acquired DNA from other organisms present in their environment [1]. Therefore, the evolution of bacterial genomes cannot be represented by trees alone but rather must be represented by more complex structures such as rhizomes illustrating the various, multiple sources of DNA that have been combined in one particular bacterial species [2]. Therefore, to a certain extent, a bacterial genome sheds light on the particular environment in which that bacterium's ancestors used to live and on the amount of DNA exchange with neighbor organisms [3]. Accordingly, genome sequencing revealed that contrary to previous conjecture, current *Mycobacterium* organisms are the result, in part, of horizontal genetic transfer from unidentified Eukarya and from environmental alpha- and gamma-Proteobacteria and Actinobacteria, as demonstrated for *Mycobacterium tuberculosis* [4]–[7]. However, the places in which *Mycobacterium* ancestors came in contact with other organisms for these genetic transfer events remained unknown.

Recent studies have shown that free-living protozoa, amoebae in particular, are indeed places in which horizontal genetic transfer occurs [8]. Free-living amoebae host numerous amoeba-resistant bacteria [3], [9]–[13], fungi [14], giant DNA viruses [15] and virophages [16], all of which live in sympatry in the free-living protozoa. Moreover, free-living protozoa are “melting pots" in which microorganisms exchange DNA including genes by horizontal gene transfer (HGT) [3], [17]–[19], as illustrated for *Rickettsia bellii* [20], *Candidatus Amoebophilus* asiaticus [21] and the recently found transfer of a *Acanthamoeba polyphaga* Mimivirus protein to *Legionella pneumophila* [22]. DNA can also be transferred from the protozoa themselves to the microorganisms, as in the cases of the *A. polyphaga* Mimivirus [15], [23], *Legionella drancourtii* [22], [24] and *Chloroflexus aurantiacus* [25]. Genetic transfers can also occur in the reverse direction, from the microorganisms to free-living protozoa, as in the case of *Tetrahymena thermophila*, which acquired bacterial genes involved in the catabolism of complex carbohydrates, contributing largely to its capacity to colonize the rumen [26]. There have also been documented transfers from bacteria to animals [27].

Non-tuberculous mycobacteria share aquatic and terrestrial ecological niches with free-living protozoa including ciliates, flagellates and amoebae [19], [28]–[30]. Co-culture experiments further showed that non-tuberculous mycobacteria could be phagocytosed by the ciliate *Tetrahymena pyriformis* [28], the social amoeba *Dictyostelium discoideum* and the free-living amoeba (FLA) *Acanthamoeba polyphaga* [19], [31]–[33] and further reside in amoebal cysts, which act as a “Trojan horse" for such amoeba-resistant mycobacteria [29], [33], [34]. *M. tuberculosis* complex organisms can also be phagocytosed by amoebae [35]–[37], and it was recently observed that, except for *Mycobacterium canetti*, *M. tuberculosis* complex members can also reside within amoebal cysts [37].

We speculated that free-living protozoa may have been places in which gene transfers into mycobacteria occurred. We performed extensive bioinformatics comparisons of available mycobacteria genomes with those of amoeba-resistant bacteria and free-living protozoa to test this hypothesis, and we used co-culture experiment to confirm its biological relevance.

## Materials and Methods

### Bacterial genome sequences and homologous gene determination

The protein complement of *M. tuberculosis* H37Rv (NC_000962), *M. tuberculosis* CDC1551 (NC_002755), *Mycobacterium bovis* (NC_002945), *Mycobacterium avium* subsp. *hominissuis* 104 (NC_008595), *M. avium* subsp. *paratuberculosis* K10 (NC_002944), *M. avium* subsp. *avium* (NZ_ACFI00000000), *Mycobacterium intracellulare* (NZ_ABIN00000000), *Mycobacterium abscessus* (NC_010397), *Mycobacterium smegmatis* mc^2^ 155 (NC_008596), *Mycobacterium marinum* (NC_010612), *Mycobacterium ulcerans* Agy99 (NC_008611), *Mycobacterium gilvum* PYR-GCK (NC_009338), *Mycobacterium sp.* JLS (NC_009077), *Mycobacterium vanbaalenii* PYR-1 (NC_008726) and *Mycobacterium leprae* TN (NC_002677) was downloaded from the National Center for Biotechnology Information (NCBI) (http://www.ncbi.nlm.nih.gov) (Table 1).

**Table 1:** Workflow summarizing the steps followed in the identification of HGT genes in mycobacteria.

1.	The proteomes of 15 *Mycobacterium* spp., *D. discoideum* and 34 amoeba-resistant bacteria (Table S1) were downloaded from the National Center for Biotechnology Information (NCBI).
2.	Search for homologous genes of mycobacterial open reading frames (ORFs) in the genomes of *D. discoideum* and 34 amoeba-resistant bacteria using the BLASTp program from NCBI (E-value<1.10–4, similarity >30% and coverage >80%).
3.	Search for the homologous sequences of the mycobacterial ORFs found in step 2 in the NR database using BLASTp (E-value<1.10–4, similarity >30% and coverage >80%).
4.	Selection of ORFs from mycobacteria found in step 3 presenting significant homology with *D. discoideum* and 34 amoeba-resistant bacteria in the first 100 hits.
5.	Infer phylogenetic relationships between the protein sequences found in steps 4 using MUSCLE for alignment and two construction methods (Maximum Likelihood within the PHYML program and M. Bayes).
6.	Analysis of the trees generated in step 5, looking for possible HGT between *Mycobacterium* spp. and amoebae and/or amoeba-resistant bacteria.

More details can be found in the materials and methods.

Each mycobacterial open reading frame (ORF) was then compared with the complete genomes of *D. discoideum* (NC_007087-92) and 34 amoeba-resistant bacteria [38] (Table S1) using the BLASTp program. The 100 hit sequences exhibiting a significant alignment (E-value<1.10^−4^) and a hit sequence with coverage ≥80% and similarity ≥30% were selected for further phylogenetic analyses. The conserved domains of selected ORFs were searched with InterProScan (http://www.ebi.ac.uk/Tools/InterProScan).

### Phylogenetic analysis and molecular data

For each set of 100 hits, the amino acid sequences were aligned using MUSCLE algorithm [39]. The alignments produced were then manually refined in order to remove regions that contain gaps or are highly divergent with the BioEdit program v7.0.9 [40].

The corrected alignments were then used for maximum likelihood (ML) and Bayesian inference (BI). ML was constructed using PHYML [41] in the PHYLIP package version 3.5c with 100 and 1,000 randomizations of input order. The substitution model was set to WAG and enabled the optimization options for tree topology, branch lengths, and rate parameters. To test the robustness of inferred topologies, posterior probabilities were determined by a Bayesian Markov chain Monte Carlo (MCMC) method implemented in the program MR BAYES V3.0 [42]. One million generations were run using the WAG matrix and model parameters (gamma shape and proportion invariant), and the trees were sampled every 100 generations. The posterior probability stabilized after 100,000 generations, so all parameter estimates before generation 100,000 were omitted. The tree with maximum posterior probability was assessed using a consensus of the final 100 000 trees. Bootstrap support of >75% and posterior probability of >90% were considered to identify supported nodes.

Substitution rates were calculated by dating the nodes in the 16S rRNA gene sequence-based phylogeny. Distances or numbers of substitutions per site separating pairs of species were estimated from the absolute numbers of differences between pairs of nucleotide sequences. We converted these data into measures of time divergence using the constant rate of 16S rRNA divergence of 0.01–0.02 per 50 million years found by Moran *et al.* [43]. All distance calculations were based on the same 1,440 sites, for which there were no missing data.

The species tree of mycobacteria was constructed based on the 16S rRNA gene sequences. The 16S rRNA sequences from the 15 studied *Mycobacterium* spp. were retrieved from NCBI database and aligned using MUSCLE. The phylogenetic relationships were inferred using the Neighbor-joining method.

### Co-culture experiments

The *A. polyphaga* Linc-AP1 strain (a gift from T. J. Rowbotham, Public Health Laboratory, Leeds, United Kingdom) was grown at 28°C for 3 days in 150-cm^3^ culture flasks (Corning, New York, USA) containing 30 ml of peptone-yeast extract-glucose (PYG) broth [44]–[46]. When the average amoeba concentration reached 5×10^5^ cells/ml, amoebae were centrifuged at 500 g for 10 min, and the pellet was suspended twice in 30 ml of Page's modified Neff's amoeba saline (PAS) (solution A-NaCl 1.20 g; MgSO_4_.7H_2_0 0.04 g; Na_2_HPO_4_ 1.42 g; KH_2_PO_4_ 1.36 g/100 ml of glass distilled water; solution B-CaCl_2_.2H_2_O 0.04 g/100 ml of distilled water; amoeba saline, 10 ml of solution A+10 ml of solution B+980 ml distilled water) [44], [46], [47]. Liquid medium-cultured *M. avium* subsp. *avium* CIP104244^T^ [33] and *L. pneumophila* strain Lens [12] organisms were washed two times with sterile phosphate-buffered saline (PBS), and the pellet was suspended in PAS. This inoculum was vortexed to minimize mycobacterial clumping. Ten milliliters of the amoebal suspension in PAS (∼10^5^ amoeba/ml) was inoculated with ∼10^6^ *L. pneumophila*/ml or ∼10^6^ *M. avium*/ml (MOI = 10) or co-infected with both bacteria. As controls, *A. polyphaga*, *L. pneumophila* and *M. avium* were cultured separately in PAS. After a 3-h incubation at 32°C, the coculture was washed three times with PAS to remove any remaining extracellular or adherent mycobacteria, and it was incubated in 10 ml PAS for 5 days at 32°C. At 0, 3 and 5 days of co-culture, *A. polyphaga* monolayers were lysed with 0.1% sodium dodecyl sulfate (SDS) (Sigma-Aldrich Logistic Gmbh, Lyon, France) for 30 min and passed through a 26-gauge needle to ensure complete lysis of the amoebae. The lysate (100 µl) was plated onto 7H10 agar for *M. avium* or Buffered Charcoal Yeast Extract (BCYE) agar plates for *L. pneumophila* and incubated for 5 to 15 days at 35 or 37°C to determine the number of colonies (CFU) of intracellular *M. avium* and *L. pneumophila*. All experiments were performed in triplicate.

### Statistical analyses

All statistical analyses mentioned in this study were performed using the chi2-square test with a significance level of p = 0.05.

## Results

### Identification of genes homologous to amoeba and amoeba-resisting bacteria in mycobacterial genomes

We searched for homologous sequences for the 65,812 ORFs of the 15 studied mycobacterial genomes in a database of free living protozoa and amoeba-resisting bacteria using a BLASTp. We found a total of 11,783 that have homologous sequences in the free living protozoa *D. discoideum* and/or amoeba-resisting bacteria (E-value<1.10–4, similarity >30% and coverage >80%). We found a total of 88 mycobacterial ORFs (0.13%) that present significant homology in the genome of the free-living protozoa *D. discoideum*. The number of ORFs with significant homology ranged from 4 genes in *M. leprae* to 29 genes in *M. smegmatis*. When comparing the 15 genomes of *Mycobacterium* spp. with the 34 available genomes of amoeba-resisting bacteria we could identify a total of 11,695 ORFs (17.8%) with significant homology in amoeba-resisting bacterial genomes. The number of mycobacterial ORFs with significant homology in the amoeba-resisting bacteria ranged from 365 for *M. leprae* to 1,208 for *M. smegmatis*. The closely related homologous genes were found in beta-Proteobacteria (30.5% ORFs), gamma-Proteobacteria (18.3% ORFs), Firmicutes (17.6% ORFs), Bacteroidetes (10.8% ORFs), delta-Proteobacteria (7.8% ORFs), Chlamydiae (6.7% ORFs) and alpha-Proteobacteria (8.3% ORFs) (Figure S1).

### Phylogenetic analyses and horizontal transfer history

**Figure 1:**
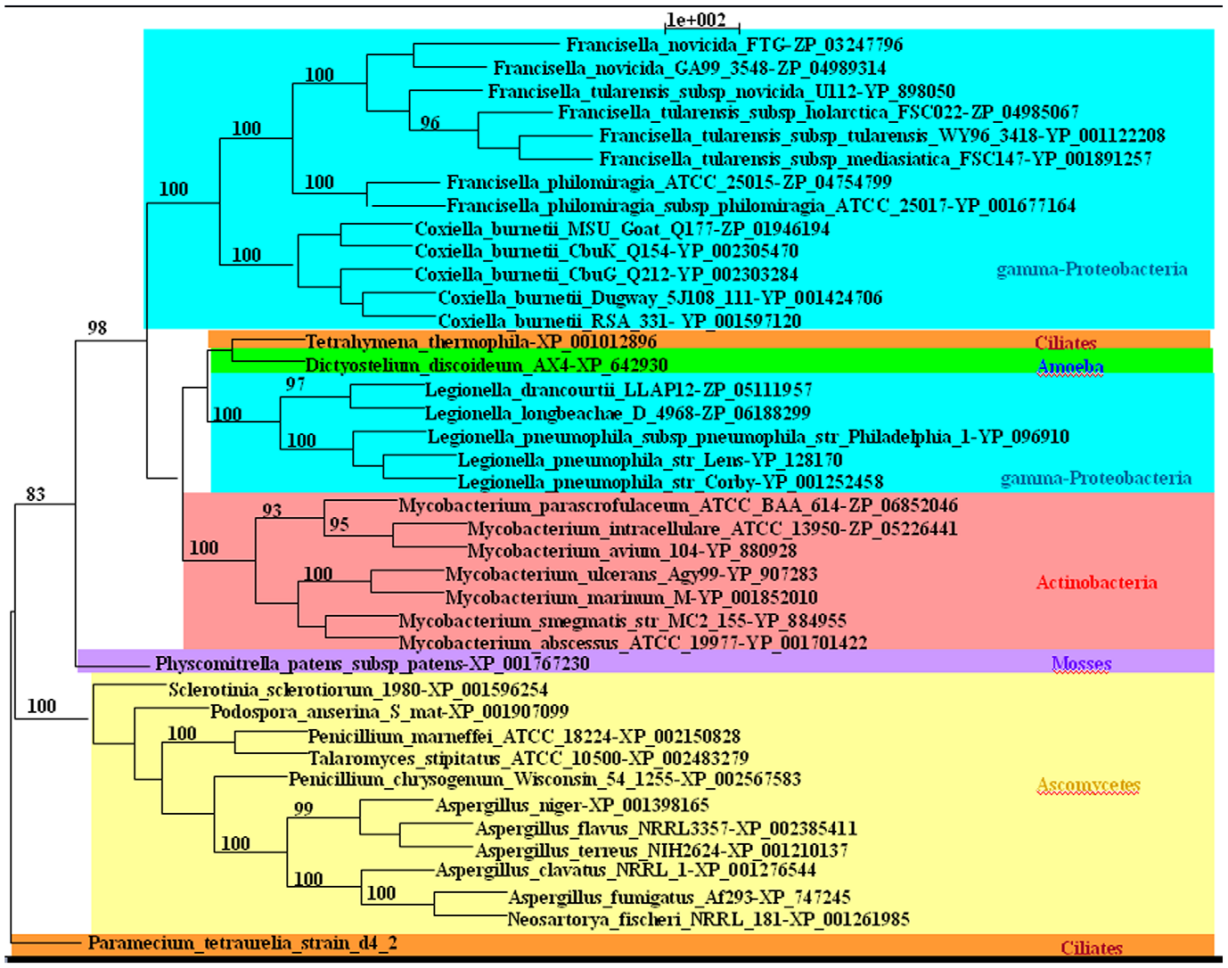
Phylogeny as inferred from the pyr-redox gene. The phylogenetic tree was obtained using maximum likelihood with the amino acid dataset. Numbers at the nodes represent bootstrap percentages. Only high bootstraps (>75) are indicated.

We searched for homologous sequences for the 11,783 ORFs in the NR database. We selected the only queries that contain free living protozoa *D. discoideum* and/or amoeba-resisting bacteria in the 100 first hits. This analysis yielded 151 sets of 100 homologous genes including sequences from free living protozoa *D. discoideum* and/or amoeba-resisting. We made 151 phylogenetic trees on the basis of these 151 gene sequences. Eight out of the 151 gene-trees showed *Mycobacterium* species in a clade with amoeba-resisting bacteria (Fig. S2, S3, S4, S5, S6, S7) and one gene (encoding for pyr-redox) showed *Mycobacterium* species in a clade with *D. discoideum* and amoeba-resisting bacteria (Fig. 1) (Table S2). Mycobacterial sequences clustered with gamma-Proteobacteria in 2/9 trees; with Archaea, gamma-Proteobacteria and Planctomyces in 1/9 trees; with Bacteroidetes and gamma-Proteobacteria in 1/9 trees; with Firmicutes spp. in 2/9 trees; with beta-Proteobacteria in 2/9 trees; and with Eukarya in 1/9 trees (Fig. S2, S3, S4, S5, S6, S7).

The gene encoding for hypothetical hydrolase placed *M. marinum* and *M. ulcerans* in a clade with *Methanosarcina acetivorans*, *Desulfovibrio salexigens*, *Planctomyces limnophilus* and *Vibrio cholerae* (Fig. S2). The gene encoding for hypothetical protein MT3512 placed *M. tuberculosis* H37Rv in a clade with *Gramella forsetii* and *Francisella tularensis* (Fig. S3). The gene encoding for amidase placed *M. marinum* in a clade with *Legionella* spp. (Fig. S4). The gene encoding for Two ORFs encoding for Acetyl CoA hydrolase in *M. marinum*, *M. ulcerans* and transcriptional regulator in *M. smegmatis*, placed these mycobacteria in clade with *Burkholderia* spp. (Fig. S5). Two ORFs encoding for sulphate transporter in *tuberculosis*, *M. bovis* and betalactamase in *M. abscessus*, placed these mycobacteria in clade with *Bacillus* spp. (Fig. S6). Finally, the gene encoding for amino acid permease placed *M. smegmatis* in a clade with *Pseudomonas putida* (Fig. S7). Further phylogenetic analyses of an ORF encoding a pyridine nucleotide disulfide oxidoreductase (pyr-redox) placed *M. marinum*, *M. ulcerans*, *M. avium*, *M. intracellulare*, *M. abscessus*, *Mycobacterium parascrofulaceum* and *M. smegmatis* in a clade with *Legionella* spp., *Francisella* spp., *Coxiella burnetii*, *T. thermophila* and *D. discoideum* with a high reliability (Fig. 1). The different construction methods showed that *Mycobacterium* spp. formed a highly supported group (bootstrap values, 94–95%) with gamma-Proteobacteria (*Legionella* spp., *C. burnetii* and *Francisella* spp.), *D. discoideum* and *T. thermophila*. In addition, we observed that *Legionella* spp. did not cluster with the other gamma-Proteobacteria but rather with *Mycobacterium* spp., *D. discoideum* (amoeba) and *T. thermophila* (ciliates) (Fig. 1). The phylogenetic construction using M. Bayes gave the same topology. The tree topology is the same when carrying out with 100 or 1,000 bootstrap replicas in what concerns the place of mycobacteria in a highly supported clade with amoeba and amoeba-resistant bacteria. Interestingly, the pyr-redox sequences matched with genes encoding for a monooxygenase with coverage of 60% and identity 25% in *Rhodococcus* and coverage of 58% and identity of 24% in *Nocardia*. These results suggest that the HGT event of pyr-redox concerns only the mycobacteria genus.

### Characteristics and functions of the horizontally transfered genes

Our findings showed that environmental mycobacteria and mycobacteria from *M. tuberculosis* complex are all affected by HGT. However, the source organisms differ between the 2 groups of mycobacteria: *M. tuberculosis* complex underwent HGT from Firmicutes, Bacteroidetes and gamma-Proteobacteria spp. while the environmental mycobacteria acquired their 7 ORFs from Firmicutes, beta- and gamma-Proteobacteria, Archaea and Eukarya (Table S2).

The nine transferred genes identified here account for 0.02–0.09% of the mycobacterial genomic content. From the nine HGT, four candidates encode for proteins involved in metabolism and five genes encode for proteins involved in information storage and processing (Table S2). Among the five genes encoding for information storage and processing, two genes encode for amidase proteins that hydrolyse the CO-NH_2_ bond with production of NH_3_, one gene encodes for a betalactamase implicated in the bacterial resistance to beta-lactam antibiotics, one gene encodes for one transcriptional regulator and one gene encodes for a hypothetical protein, characterized by the presence of a formyl_trans_N domain and belonging to the transferase family. Among the genes encoding for metabolic proteins, two genes are implicated in transporter of different substrates across the membrane including the sulfate transporter and the amino acid permease and two genes encode for the Acetyl-CoA hydrolase and a pyridine nucleotide disulfide oxidoreductase.

The gene length of these ORFs varies from 702 to 1,485 pb. These ORFs are widely distributed across the genomes of *Mycobacterium* spp. The detailed observation of the regions surrounding these HGT candidates, i.e. 10 genes upstream and downstream, revealed the presence of 4 transposases in 3 mycobacterial genomes *M. ulcerans*, *M. smegmatis* and *M. avium* (Table S3). The GC content of 5 transferred genes significantly differ from the GC content of the genome in *M. tuberculosis*, *M. bovis*, *M. smegmatis*, *M. ulcerans* and *M. marinum* (p<0.05) (Table S3). Only 2 out of 9 transfered genes present both a GC% significantly differing from that of the mycobacterial host genome and transposase gene in the close vicinity.

### Co-culture experiments

**Figure 2:**
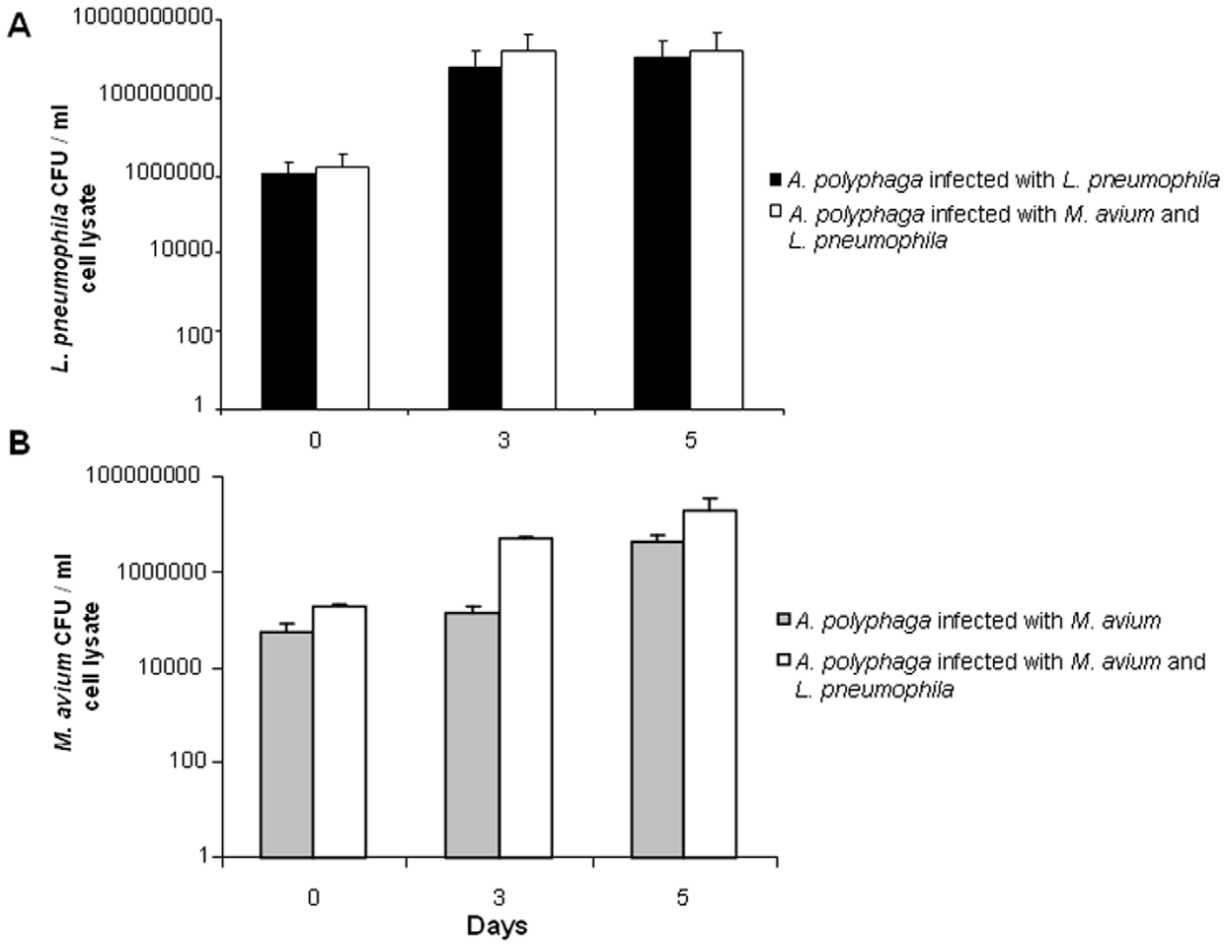
*A. polyphaga* co-cultured with *M. avium* and *L. pneumophila* for 5 days. A) The number of *L. pneumophila* colonies was obtained after plating the lysate of *L. pneumophila* and *A. polyphaga* culture or *L. pneumophila*, *M. avium* and *A. polyphaga* co-culture in BCYE agar medium. B) The number of *M. avium* colonies was obtained after plating the lysate of *M. avium* and *A. polyphaga* culture or *M. avium*, *L. pneumophila* and A. polyphaga co-culture in 7H10 agar medium. Data points are the means of triplicate wells, and the standard errors are represented by error bars.

We co-cultured the amoeba *A. polyphaga* with both *L. pneumophila* and *M. avium*, and we observed that *L. pneumophila and M. avium* could indeed live together in amoebae for at least five days. We first observed that the number of *A. polyphaga* trophozoites infected with *M. avium*, *L. pneumophila* or both strains increased significantly (p≤0.05) over the course of the experiments. The quantification of the colony forming units (CFU) of *M. avium* and *L. pneumophila* when co-cultured with amoebae yielded 1.66×10^6^±1.68×10^5 ^CFU/mL at day 0, 1.52×10^9^±2.5×10^8 ^CFU/mL at day 3 and 1.65×10^9^±2.76×10^8 ^CFU/mL at day 5 for *L. pneumophila* and 1.99×10^5^±1.63×10^4 ^CFU/mL at day 0, 5.2×10^6^±7.07×10^5 ^CFU/mL at day 3 and 2.05×10^7^±1.48×10^7 ^CFU/mL at day 5 for *M. avium* (Fig. 2).

## Discussion

Our phylogenetic analyses identified eight mycobacterial genes that have close phylogenetic relationships with bacteria other than *Actinobacteria* spp. Given that most of these species are amoeba-resistant, the phylogenies were highly suggestive of possible HGT within amoeba. Nonetheless, the lack of information about the direction of the transfer hampered the elucidation of the HGT history. Furthermore, we found one gene encoding for pyr-redox that gave insight into the history of the HGT events in relation with the mycobacterial lifestyle within free-living protozoa.

It has been previously shown that the *Mycobacterium* spp. and gamma-Proteobacteria studied herein are able to live alone in amoebae [9], [29], [38], [48] as well as in ciliates [12] or together in *Acanthamoeba castellanii* [49]. We therefore co-cultured the amoeba *A. polyphaga* with both *L. pneumophila* and *M. avium*, and we observed that *L. pneumophila* and *M. avium* could indeed live together in amoebae for at least five days. Thus, our data expand the previous demonstration of intra-amoebal surviving of both *Legionella* and mycobacteria in amoeba *A. castellanii* to another species of amoeba, *A. polyphaga*. This sympatric lifestyle, *i.e.*, various microorganisms living together, provides opportunities for DNA exchange and gene transfer within amoebae [3], [15], [23]. This hypothesis agrees with the current model for the evolution of mycobacteria, which postulates that the ancestor of mycobacteria was an environmental organism living in an aquatic habitat [50]. Recent genome analysis of the environmental *Mycobacterium indicus pranii*, a member of the *M. avium* complex, further supports this hypothesis in which the most recent common ancestor of mycobacteria gave rise to waterborne *M. marinum* and *M. ulcerans* on one branch, the *M. avium* complex on a second branch and the *M. tuberculosis* complex on a third branch [50].

Life in free-living amoebae has been demonstrated to protect amoeba-resistant organisms, such as environmental mycobacteria and *Legionella*, against adverse environmental conditions [3], [9], [38], [48], to increase their resistance to some antibiotics [48], [51], [52] and to enhance their virulence [36], [48], [51]. The pyr-redox gene studied herein is present in *Mycobacterium* spp. that have been shown to survive in amoebal cysts. The significant association between the presence of pyr-redox and the survival in amoebal cysts (chi2-square, p = 0.002) highlights the possible role of this protein in the intraamoebal lifestyle and life inside macrophages [19], [48]. During phagocytosis, amoebae and macrophages produce the oxygen metabolites nitric oxide and hydrogen peroxide, which generate a toxic environment that can kill phagocytized bacteria [53]–[55]. *Mycobacterium* spp. deploy multiple strategies to resist to this oxidative stress, including the expression of catalase/peroxidase [56] and superoxide dismutase [57], a thiol-based detoxification response [58] and the pyr-redox response [59], [60]. Pyr-redox complements the anti-oxidative arsenal of mycobacteria during their survival in amoebae and macrophages.

**Figure 3:**
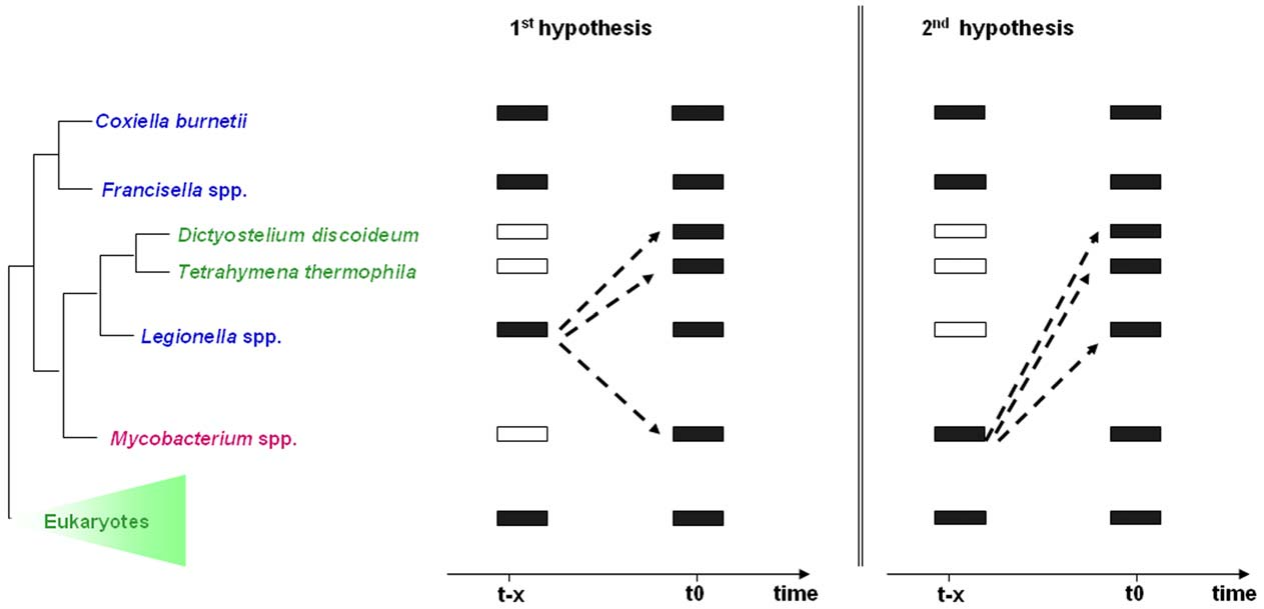
Schematic representation of two alternative explanations of the evolutionary history of the pyridine nucleotide disulfide oxidoreductase gene. Dashed arrows indicate the possible lateral transfer of the gene. First hypothesis: The gene encoding pyr-redox exists in the gamma-Proteobacteria species and is absent in *D. discoideum* and *T. thermophila*. This gene was acquired from *Legionella* spp. by *Mycobacterium* spp., *D. discoideum* and *T. thermophila*. Second hypothesis: The gene encoding pyr-redox was lost from *Legionella* spp., *D. discoideum* and *T. thermophila* and was acquired later from *Mycobacterium* spp. T0 corresponds to the time of observation and T-x to the time when the event occurred.

Phylogenetic trees have indicated that phylogenetically distant organisms have acquired the pyr-redox via HGT, but the source of this transfer is ambiguous (Fig. 3). According to one scenario, pyr-redox was acquired by *Mycobacterium* spp., *D. discoideum* and *T. thermophila* from gamma-Proteobacteria, specifically from *Legionella* spp. (Fig. 3). This result agrees with previously published observations that genes acquired by HGT in Actinobacteria mostly originated from beta- and gamma-Proteobacteria [6]. The genes acquired by HGT in fungi [61] and HGT in ciliates such as *T. thermophila* [26], [62] mostly originated from bacteria. *D. discoideum* may have transferred the pyr-redox gene to *Mycobacterium* spp. or may have been the place for transfer. According to a second scenario, there were multiple gene losses in *Legionella* spp., *D. discoideum* and *T. thermophila* and recent acquisitions from *Mycobacterium* spp. (Fig. 3). Whereas several studies have demonstrated HGT between mycobacteria [63], HGT originating from *Mycobacterium* spp. has never been reported. Considering the paraphyly of *Tetrahymena* spp. and *Dictyostelium* spp., we have to postulate a minimum of two independent HGT events in both scenarii: the first one event from *Legionella* spp. or mycobacteria (ancestors) into amoebae and amoeba-resistant bacteria and the second event from *Legionella* spp. or mycobacteria (ancestors) into *T. thermophila.* Alternatively, the scenarii might have required a single ancient HGT event in a certain common ancestor of eukaryotes and subsequent multiple losses from organisms except *T. thermophila* and *D. discoideum.*

**Figure 4:**
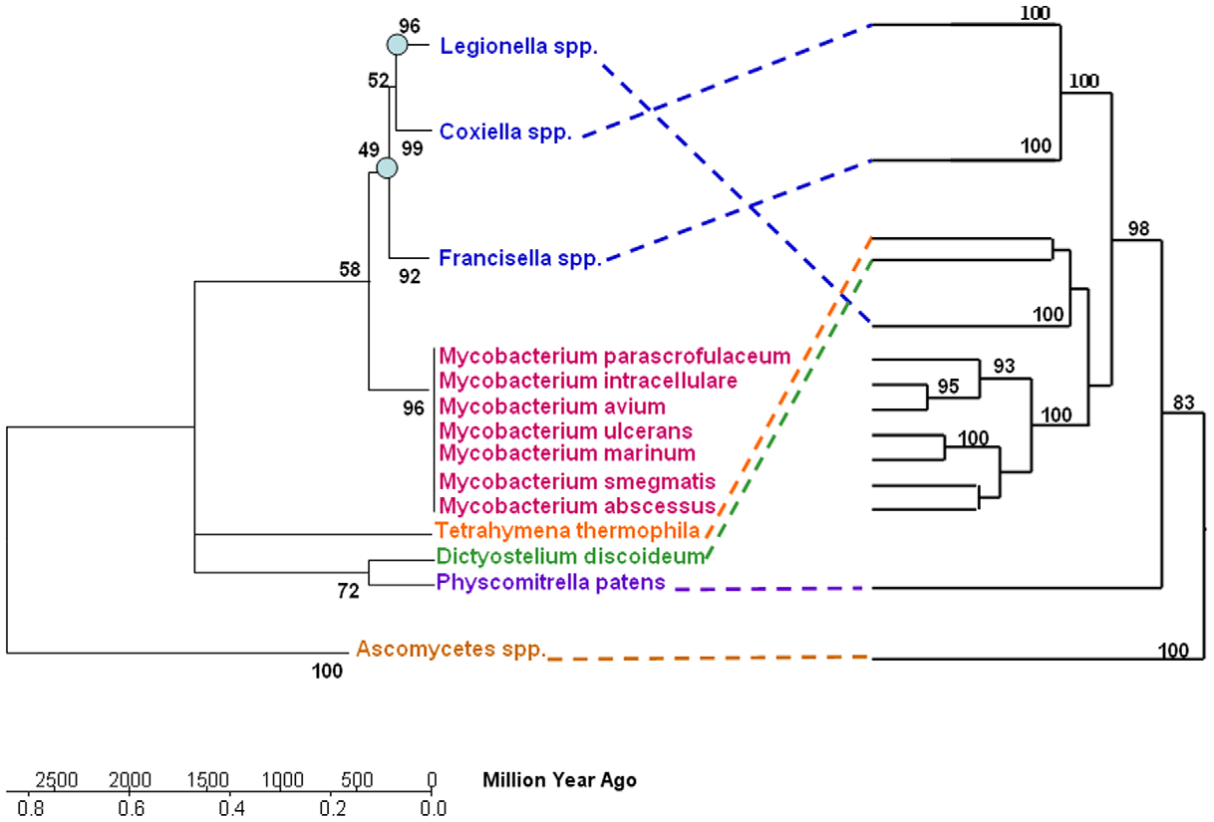
Schematic representation of HGT and molecular clock. The left tree shows the relationships and approximate dates of divergence of the species. The phylogeny was reconstructed based on 16S and 18S rDNA sequences, and the expected divergence was calculated as a function of time, 1–2% per 50 MYA. The right tree shows the relationships among species based on pyr redox protein. The circles on the nodes indicate the radiation of *Legionella* spp. (33 MYA) and the separation of gamma-Proteobacteria spp. (267 MYA).

Both scenarii involve HGT from a bacterium to a eukaryote (*Tetrahymena* spp. and *Dictyostelium)* following the loss of the eukaryotic pyr-redox gene from these genomes. Indeed, the gene encoding pyr-redox might have become disused or lost its functional importance, allowing the loss of the gene. The second scenario requires additional losses from *Legionella* genomes that occurred before the HGT and is less parsimonious than the first one. Thus, the scenario that postulates HGT from *Legionella* or *Dictyostelium* into mycobacteria seems to be more likely. The molecular clock showed that mycobacteria and amoeba-resistant gamma-Proteobacteria exchanged the pyr-redox gene between 33 and 267 Million Years Ago, after the separation of gamma-Proteobacteria spp. and before the radiation of *Legionella* spp. (Fig. 4). This range provides an estimated time-frame for the intracellular association of mycobacteria within amoebae and subsequent horizontal gene transfers. The pyr-redox gene has been found in 5/21 annotated *Mycobacterium* genomes, with the notable exception of the *M. tuberculosis* complex members. The genome of *M. tuberculosis* has been shown to exhibit the highest ratio of eukaryotic-prokaryotic gene fusion [64], but this observation was made before protist genomes were sequenced. The most parsimonious scenario suggests that the pyr-redox gene was acquired by an ancestor of all mycobacterial species, followed by a loss by the *M. tuberculosis* complex members *M. leprae*, *M. vanbaalenii*, *M. gilvum* and *Mycobacterium* sp. JLS.

In conclusion, our phylogenetic analyses found 8 ORFs most likely acquired through HGT in *Mycobacterium* spp. and one further pyr-redox ORF that elucidates the history of the HGT events in relation with the mycobacterial lifestyle within free-living protozoa. The experimental data reported herein support these genome-based analyses. Amoebae or other phagocytic organisms may have been the places in which the gene exchanges occurred. Thus, *Mycobacterium* spp. have followed an evolutionary strategy similar to that of other intracellular bacteria: they interfere with host cellular processes through the expression of genes horizontally acquired from the host. HGT may have contributed to the adaptation of mycobacteria to an intracellular lifestyle.

## Supporting Information

Figure S1 **Putative sources of homologous ORFs from bacteria other than Actinobacteria in the mycobacterial genome.**
(PDF)

Figure S2 **Extended phylogenetic tree showing representatives of the conserved hypothetical hydrolase.** Phylogenetic trees showing HGT events as generated by the Maximum Likelihood method. Numbers at nodes are bootstrap percentages based on 100 resamplings. The scale bar represents the number of estimated changes per position for a unit of branch length. *Mycobacterium* spp. are colored in red.
(PDF)

Figure S3 **Extended phylogenetic tree showing representatives of hypothetical protein MT3512.** Phylogenetic trees showing HGT events as generated by the Maximum Likelihood method. Numbers at nodes are bootstrap percentages based on 100 resamplings. The scale bar represents the number of estimated changes per position for a unit of branch length. *Mycobacterium* spp. are colored in red.
(PDF)

Figure S4 **Extended phylogenetic tree showing representatives of amidase.** Phylogenetic trees showing HGT events as generated by the Maximum Likelihood method. Numbers at nodes are bootstrap percentages based on 100 resamplings. The scale bar represents the number of estimated changes per position for a unit of branch length. *Mycobacterium* spp. are colored in red.
(PDF)

Figure S5 **Extended phylogenetic tree showing representatives of A) acetyl CoA hydrolase and B) transcriptional regulator.** Phylogenetic trees showing HGT events as generated by the Maximum Likelihood method. Numbers at nodes are bootstrap percentages based on 100 resamplings. The scale bar represents the number of estimated changes per position for a unit of branch length. *Mycobacterium* spp. are colored in red.
(PDF)

Figure S6 **Extended phylogenetic tree showing representatives of A) sulfate transporter and B) beta-lactamase.** Phylogenetic trees showing HGT events as generated by the Maximum Likelihood method. Numbers at nodes are bootstrap percentages based on 100 resamplings. The scale bar represents the number of estimated changes per position for a unit of branch length. *Mycobacterium* spp. are colored in red.
(PDF)

Figure S7 **Extended phylogenetic tree showing representatives of amino acid permease.** Phylogenetic trees showing HGT events as generated by the Maximum Likelihood method. Numbers at nodes are bootstrap percentages based on 100 resamplings. The scale bar represents the number of estimated changes per position for a unit of branch length. Mycobacterium spp. are colored in red.
(PDF)

Table S1 **Genome sequence of amoeba-resistant bacteria utilized in this study.**
(DOC)

Table S2 **Genes probably transferred by horizontal gene transfer.**
(DOC)

Table S3 **Description of the nine probably transfered genes.**
(DOC)
