# Supplementary figures and images for: The Genealogic Tree of Mycobacteria Reveals a Long-Standing Sympatric Life into Free-Living Protozoa
Source: PLoS One. 2012 Apr 12;7(4):e34754. doi: 10.1371/journal.pone.0034754 (PMC3325273; doi:10.1371/journal.pone.0034754)

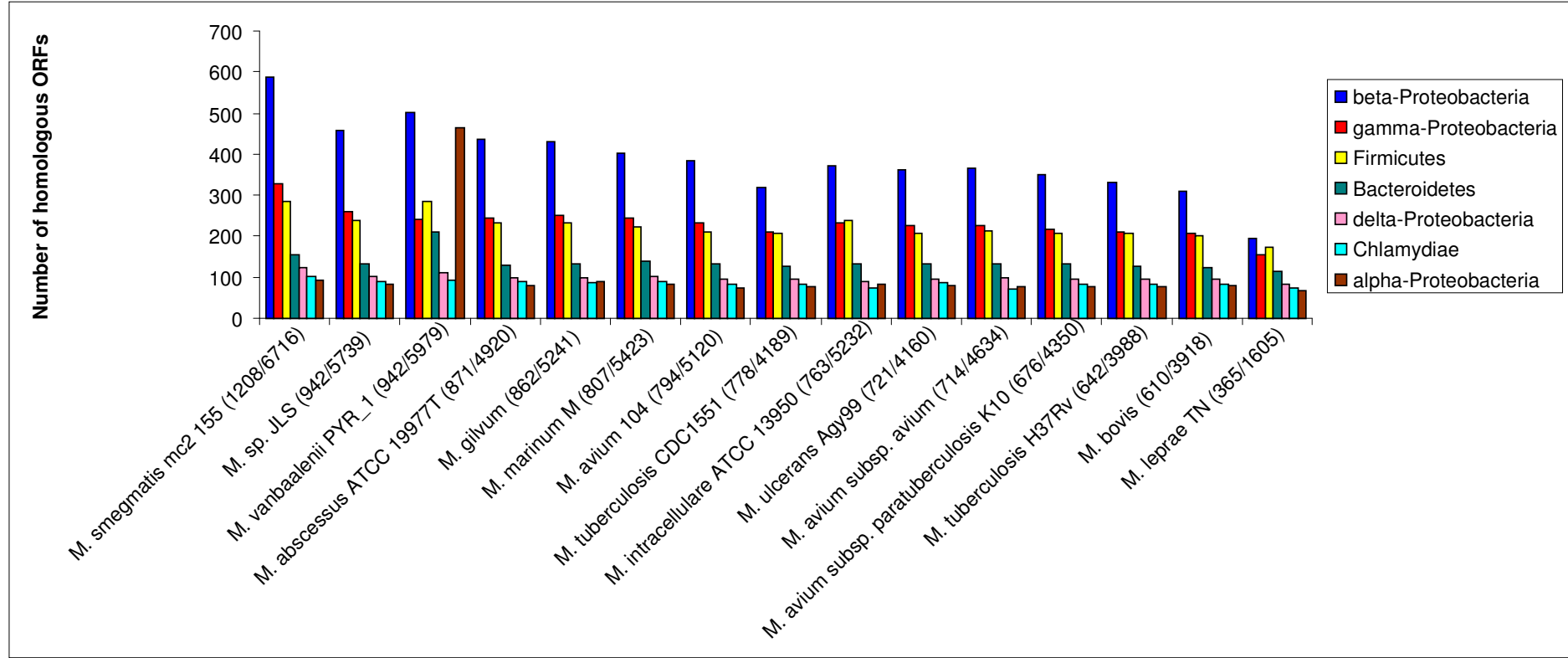

Supplement: Figure S1 — Putative sources of homologous ORFs from bacteria other than Actinobacteria in the mycobacterial genome. (PDF) [file pone.0034754.s001.pdf]

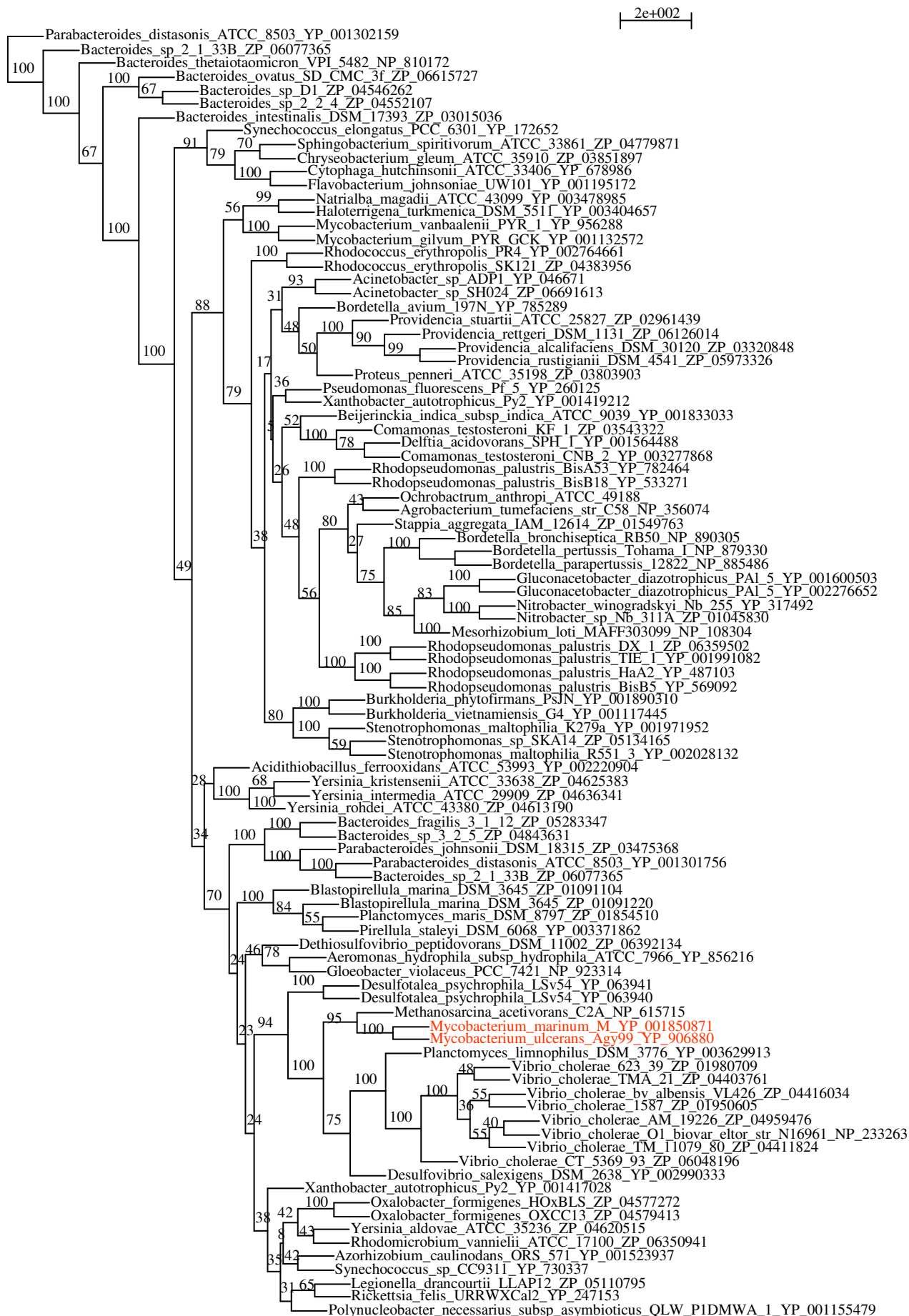

Supplement: Figure S2 — Extended phylogenetic tree showing representatives of the conserved hypothetical hydrolase. Phylogenetic trees showing HGT events as generated by the Maximum Likelihood method. Numbers at nodes are bootstrap percentages based on 100 resamplings. The scale bar represents the number of estimated changes per position for a unit of branch length. Mycobacterium spp. are colored in red. (PDF) [file pone.0034754.s002.pdf]

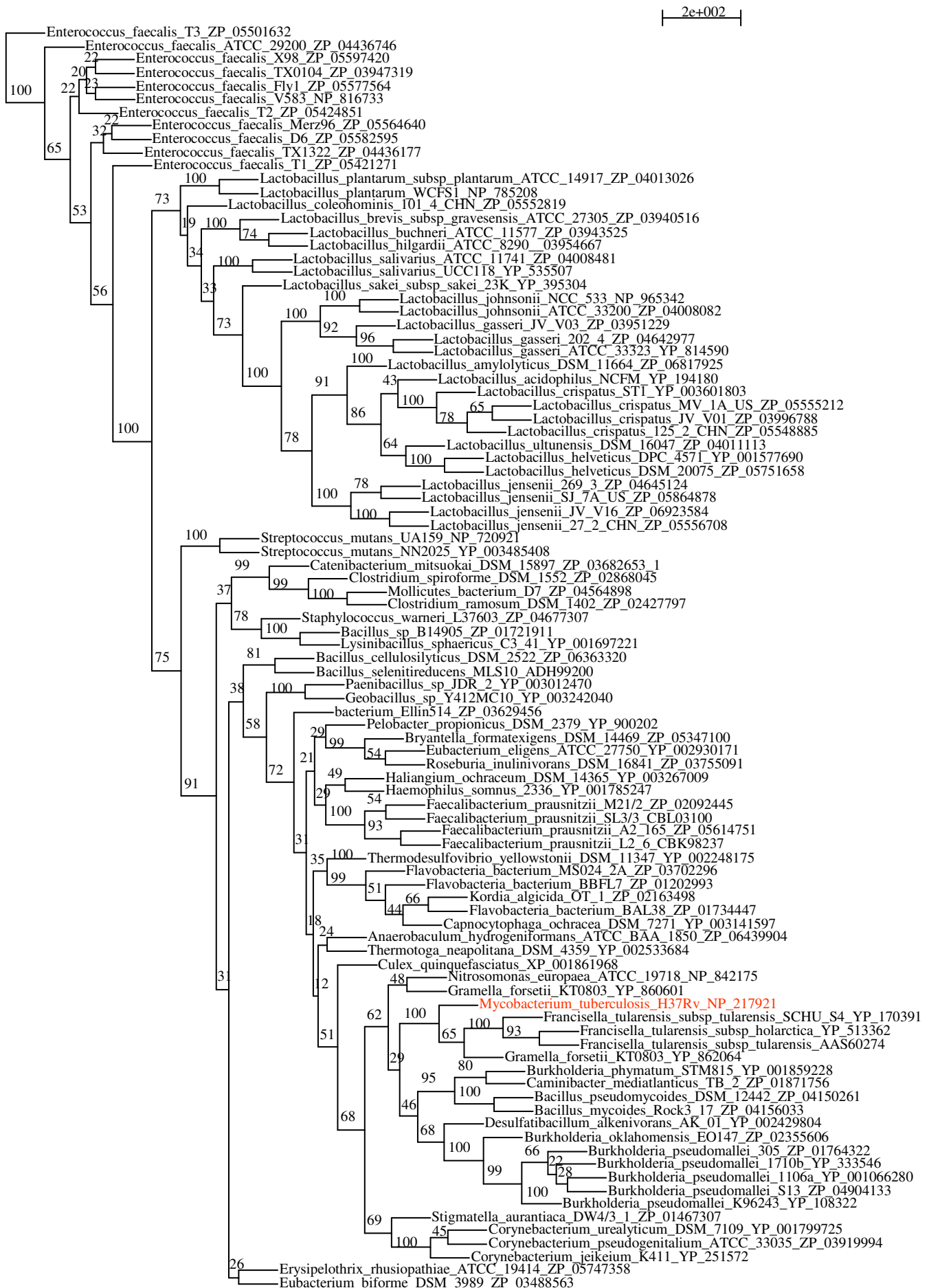

Supplement: Figure S3 — Extended phylogenetic tree showing representatives of hypothetical protein MT3512. Phylogenetic trees showing HGT events as generated by the Maximum Likelihood method. Numbers at nodes are bootstrap percentages based on 100 resamplings. The scale bar represents the number of estimated changes per position for a unit of branch length. Mycobacterium spp. are colored in red. (PDF) [file pone.0034754.s003.pdf]

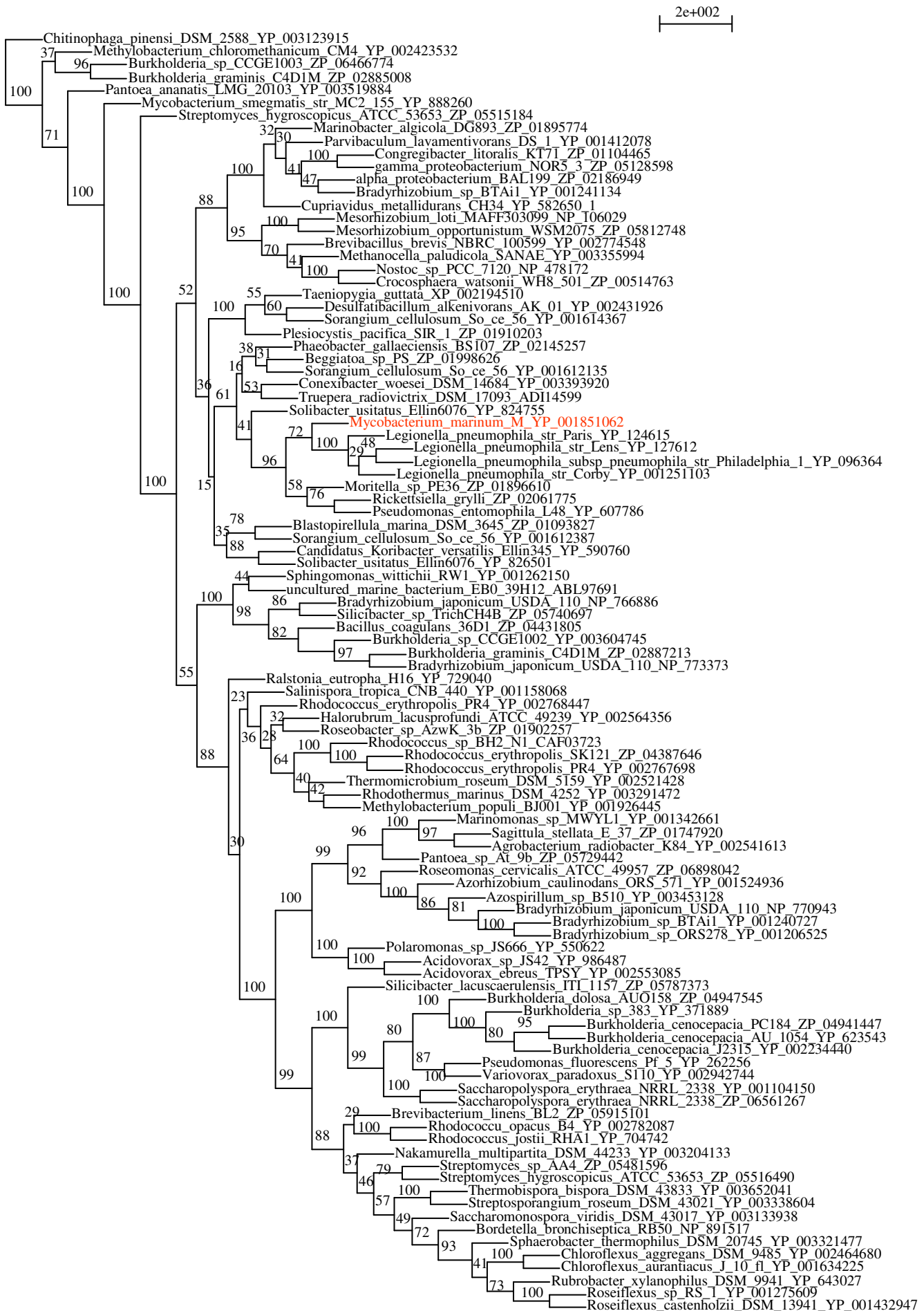

Supplement: Figure S4 — Extended phylogenetic tree showing representatives of amidase. Phylogenetic trees showing HGT events as generated by the Maximum Likelihood method. Numbers at nodes are bootstrap percentages based on 100 resamplings. The scale bar represents the number of estimated changes per position for a unit of branch length. Mycobacterium spp. are colored in red. (PDF) [file pone.0034754.s004.pdf]

# A) Acetyl CoA hydrolase

2e+002

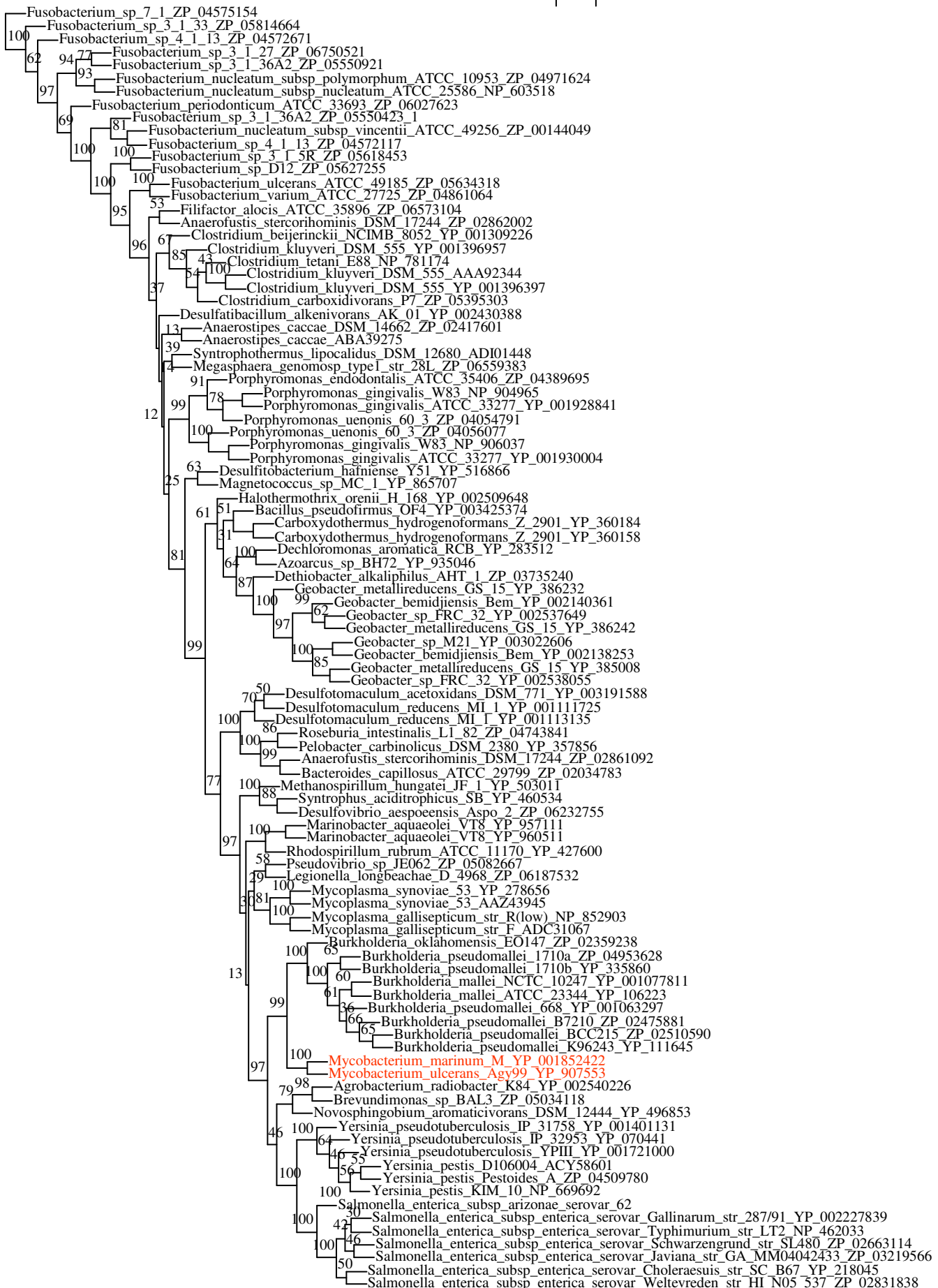

## B) Transcriptional regulator

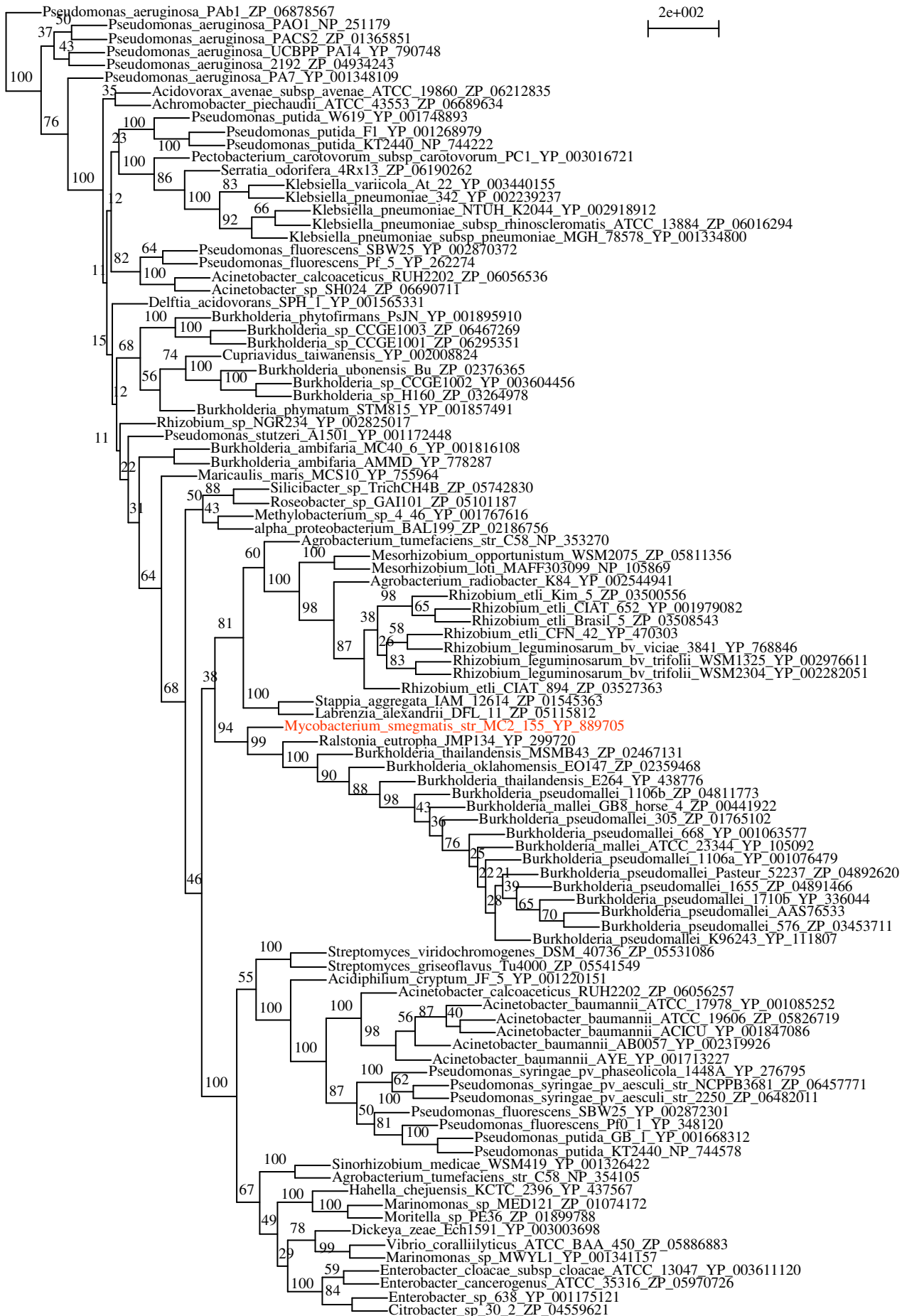

Supplement: Figure S5 — Extended phylogenetic tree showing representatives of A) acetyl CoA hydrolase and B) transcriptional regulator. Phylogenetic trees showing HGT events as generated by the Maximum Likelihood method. Numbers at nodes are bootstrap percentages based on 100 resamplings. The scale bar represents the number of estimated changes per position for a unit of branch length. Mycobacterium spp. are colored in red. (PDF) [file pone.0034754.s005.pdf]

# A) Sulfate transporter

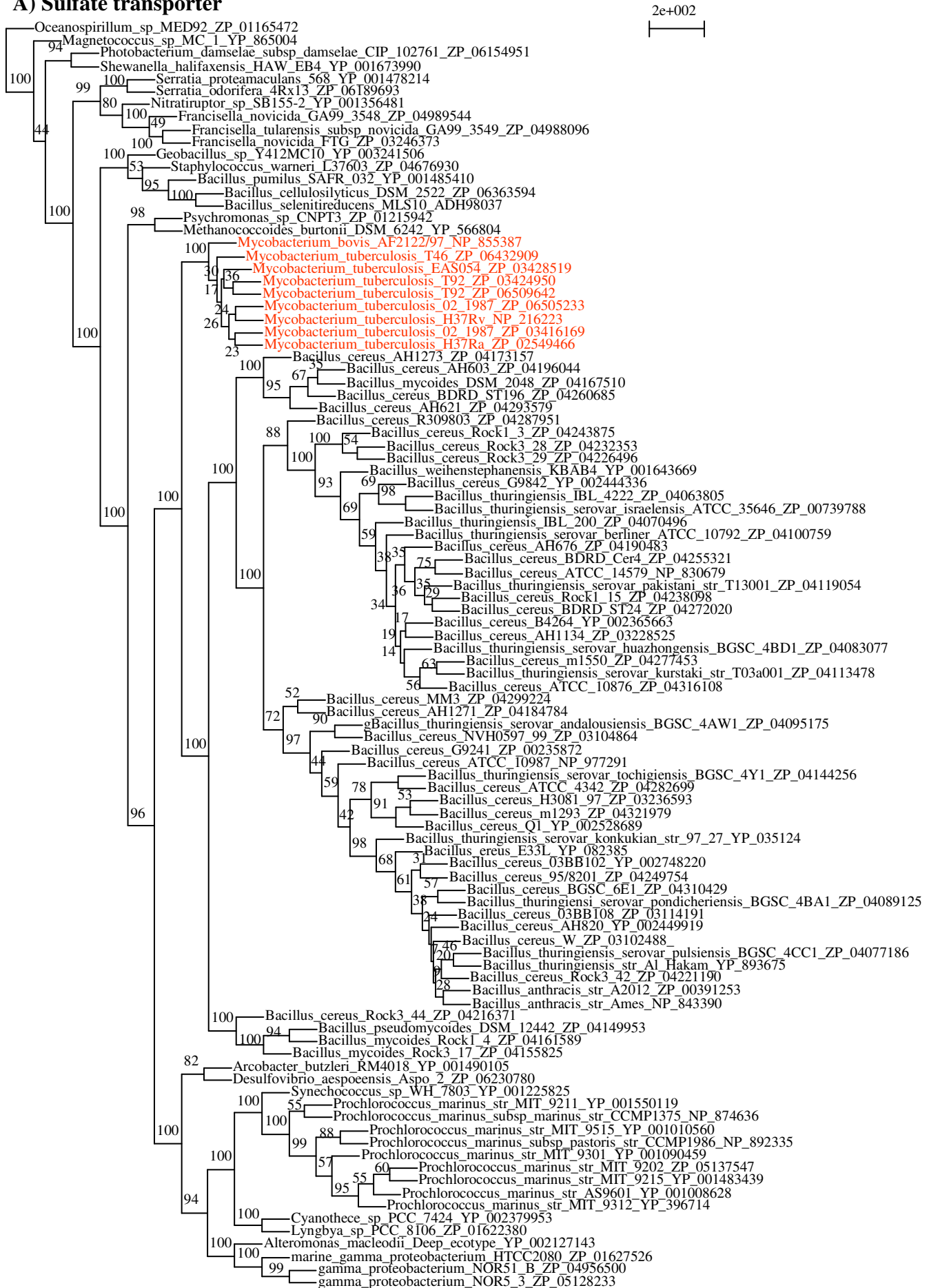

## B) Betalactamase

2e+002

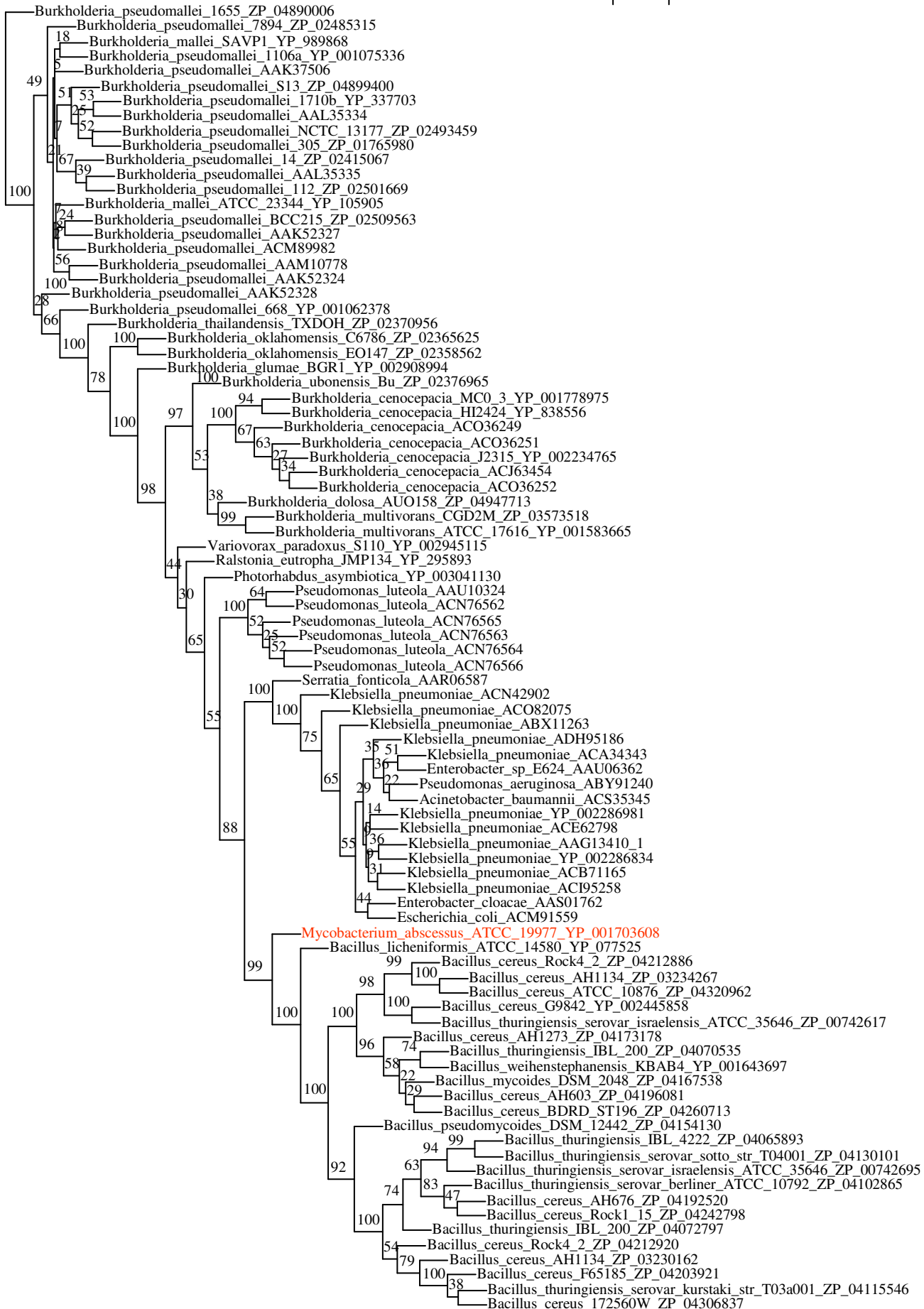

Supplement: Figure S6 — Extended phylogenetic tree showing representatives of A) sulfate transporter and B) beta-lactamase. Phylogenetic trees showing HGT events as generated by the Maximum Likelihood method. Numbers at nodes are bootstrap percentages based on 100 resamplings. The scale bar represents the number of estimated changes per position for a unit of branch length. Mycobacterium spp. are colored in red. (PDF) [file pone.0034754.s006.pdf]

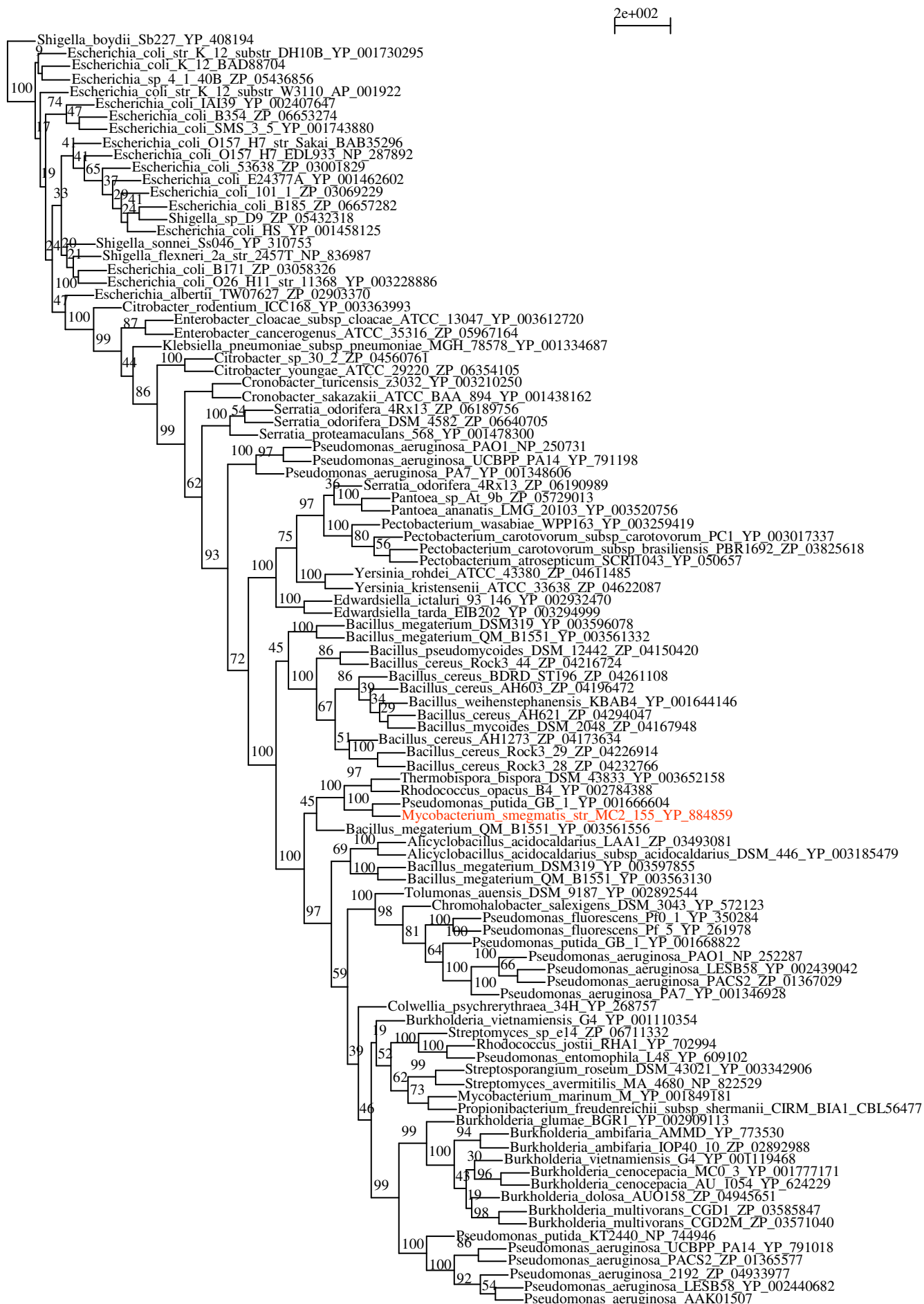

Supplement: Figure S7 — Extended phylogenetic tree showing representatives of amino acid permease. Phylogenetic trees showing HGT events as generated by the Maximum Likelihood method. Numbers at nodes are bootstrap percentages based on 100 resamplings. The scale bar represents the number of estimated changes per position for a unit of branch length. Mycobacterium spp. are colored in red. (PDF) [file pone.0034754.s007.pdf]
