# Supplementary material for: The Genealogic Tree of Mycobacteria Reveals a Long-Standing Sympatric Life into Free-Living Protozoa
Source: PLoS One. 2012 Apr 12;7(4):e34754. doi: 10.1371/journal.pone.0034754 (PMC3325273; doi:10.1371/journal.pone.0034754)
Supplement: Table S1 — Genome sequence of amoeba-resistant bacteria utilized in this study. (DOC) [file pone.0034754.s008.doc]

Table S1. Genome sequence of amoeba-resisting bacteria utilised in this study.

| **Bacterial species** | **Described interaction with protozoa** | **Phylum** | **Class** | **Genomes** | **Lengh (nt)** | **GC content (%)** |
| --- | --- | --- | --- | --- | --- | --- |
| *Aeromonas hydrophila* | IC survival (Ac) | Proteobacteria | γ-proteobacteria | NC_008570 | 4,744,448 | 61 |
| *Bacillus cereus* | IC multiplication (Ap) | Firmicutes | Bacilli | NC_012472 | 5,269,628 | 35 |
| *Burkholderia pseudomallei* | IC survival (As) | Proteobacteria | β-proteobacteria | NC_009076-NC_009078 | 7 890 249 | 68 |
| *Candidatus* Protochlamydia amoebophila UWE25 | IC survival and multiplication (Ap) | Chlamydiae | chlamydias | NC_005861 | 2,414,465 | 34 |
| *Campylobacter jejuni* | IC multiplication (Ap) | Proteobacteria | ε-proteobacteria | NC_009839 | 1,628,115 | 30 |
| *Campylobacter lari* | IC multiplication (Ap) | Proteobacteria | ε-proteobacteria | NC_012039 | 1,525,460 | 29 |
| *Chlamydophila pneumoniae* | IC survival (Ac) | Chlamydiae | chlamydias | NC_002179 | 1,229,853 | 40 |
| *Coxiella burnetii* | IC survival (Ac) | Proteobacteria | γ-proteobacteria | NC_011527 | 2,008,870 | 42 |
| *Edwardsiella tarda* | IC survival (Tp) | Proteobacteria | γ-proteobacteria | NC_013508 | 3,760,463 | 59 |
| *Francisella tularensis* | IC multiplication (Ac), IK survival (Ac) | Proteobacteria | γ-proteobacteria | NC_008245 | 1,892,616 | 32 |
| *Helicobacter pylori* | IC survival (Ac) | Proteobacteria | γ-proteobacteria | NC_000915 | 1,667,867 | 38 |
| *Klebsiella pneumoniae* | IC survival (Ac) | Proteobacteria | γ-proteobacteria | NC_011283 | 5,641,239 | 57 |
| *Legionella longbeachae* | IC multiplication (*Acanthamoeba* sp., Tp) | Proteobacteria | γ-proteobacteria | NC_013861 | 4,077,332 | 37 |
| *Legionella pneumophila* str. Corby | IC multiplication (>20 FLA species) | Proteobacteria | γ-proteobacteria | NC_009494 | 3,576,470 | 38 |
| *Legionella pneumophila* str. Lens | IC multiplication (>20 FLA species) | Proteobacteria | γ-proteobacteria | NC_006369 | 3,345,687 | 38 |
| *Legionella pneumophila* str. Paris | IC multiplication (>20 FLA species) | Proteobacteria | γ-proteobacteria | NC_006368 | 3,503,610 | 38 |
| *Legionella pneumophila* str. Philadelphia | IC multiplication (>20 FLA species) | Proteobacteria | γ-proteobacteria | NC_002942 | 3,397,754 | 38 |
| *Legionella drancourtii* | IC multiplication | Proteobacteria | γ-proteobacteria | NZ_ACUL00000000* | 4,067,717 | 39 |
| *Listeria monocytogenes* | IC multiplication (Tp), IC survival (Ac) | Firmicutes | Bacilli | NC_013766 | 3,032,288 | 37 |
| *Listeria seeligeri* | IC survival (Ac) | Firmicutes | Bacilli | NC_013891 | 2,797,636 | 37 |
| *Listeria welshimeri* | IC survival (Ac) | Firmicutes | Bacilli | NC_008555 | 2,814,130 | 36 |
| *Pasteurella multocida* | IC multiplication (Ap) | Proteobacteria | γ-proteobacteria | NC_002663 | 2,257,487 | 40 |
| *Porphyromonas gingivalis* | IC multiplication (Ac) | Bacteroidetes | Bacteroidia | NC_010729 | 2,354,886 | 48 |
| *Pseudomonas aeruginosa* | IC multiplication (Ap, *Echinamoeba* sp.) | Proteobacteria | γ-proteobacteria | NC_011770 | 6,601,757 | 66 |
| *Ralstonia pickettii* | IC multiplication (Ap, *Naegleria lovaniensis*) | Proteobacteria | β-proteobacteria | NC_012856-NC_012857 | 4,971,045 | 64 |
| *Rickettsia bellii* | IC survival and multiplication | Proteobacteria | α-proteobacteria | NC_009883 | 1,528,980 | 31 |

**Table S**1. Continued

| **Bacterial species** | **Described interaction with protozoa** | **Phylum** | **Class** | **Genomes** | **Lengh (nt)** | **GC content (%)** |
| --- | --- | --- | --- | --- | --- | --- |
| *Salmonella typhimurium* | IC multiplication (Ap) | Proteobacteria | γ-proteobacteria | NC_003197 | 4,857,432 | 52 |
| *Shigella dysenteriae* | IC multiplication (Ac) | Proteobacteria | γ-proteobacteria | [NC_007606](http://www.ncbi.nlm.nih.gov/nuccore/NC_007606) | 4,369,232 | 51 |
| *Shigella sonnei* | IC multiplication (Ac) | Proteobacteria | γ-proteobacteria | NC_007384 | 4,825,265 | 51 |
| *Staphylococcus aureus* (MRSA) | IC multiplication (Ap), IK survival (?) | Firmicutes | Bacilli | NC_002745 | 2,814,816 | 32 |
| *Streptococcus pneumoniae* | IC multiplication (Ap) | Firmicutes | Bacilli | NC_011900 | 2,221,315 | 39 |
| *Vibrio cholerae* | IC multiplication and IK survival (Ac, *Naegleria gruberi*) | Proteobacteria | γ-proteobacteria | NC_002505-NC_002506 | 4,003,464 | 47 |
| *Yersinia enterocolitica* | IC survival (Ac) | Proteobacteria | γ-proteobacteria | NC_008800 | 4,615,899 | 47 |
| *Yersinia petis* | IC survival (*Hartmannella rhysodes*) | Proteobacteria | γ-proteobacteria | NC_010159 | 4,504,254 | 47 |

* Genome Project sequencing in our laboratory.

Ac, *Acanthamoeba castellanii*; AP, *Acanthamoeba PolyPhaga*; Hv, *Hartmannella vermiformis*; TP, *Tetrahymena Pyriformis*; IC, intracellular; IK, intracyst
