# Supplementary material for: The Genealogic Tree of Mycobacteria Reveals a Long-Standing Sympatric Life into Free-Living Protozoa
Source: PLoS One. 2012 Apr 12;7(4):e34754. doi: 10.1371/journal.pone.0034754 (PMC3325273; doi:10.1371/journal.pone.0034754)
Supplement: Table S2 — Genes probably transferred by horizontal gene transfer. (DOC) [file pone.0034754.s009.doc]

Table S2. Genes probably transfered by horizontal gene transfer.

| **Phylum** | **Potential donor** | **Annotation of genes** | **Biologic function** | **COGs Description** | **Potential acceptor** | **Gene length (pb)** | **Localization in genome** |
| --- | --- | --- | --- | --- | --- | --- | --- |
| Firmicutes | *Bacillus cereus* | Sulfate transporter | Transport of sulfate across a membrane | Metabolism | *Mycobacterim bovis* | 1458 | 1920223-1921683 |
|  |  |  |  |  | *Mycobacterium tuberculosis* H37Rv |  | 1934882-1936342 |
|  |  | Betalactamase | Resistance of bacteria to beta-lactam antibiotics | Information storage and processing | *Mycobacterium abscessus* | 867 | 2927879-2928748 |
| beta-Proteobacteria | *Burkholderia pseudomallei* | Acetyl-CoA hydrolase | Participate in pyruvate metabolism: AcetylcoA + H2O <==> CoA + Acetate | Metabolism | *Mycobacterium marinum* | 1335 | 5121820-5123157 |
|  |  |  |  |  | *Mycobacterium ulcerans* |  | 4494715-4496052 |
|  |  | AraC family protein, transcriptional regulator | Regulation of transcription | Information storage and processing | *Mycobacterium smegmatis* | 849 | 5548969-5549820 |
| gamma-Proteobacteria | *Francisella tularensis* | Hypothetical protein MT3512 | Belong to the formyl trasnferase family. Can participate in different pathway such as methionine metabolism |  | *Mycobacterium tuberculosis* H37Rv | 702 | 3823880-3824584 |
|  | *Pseudomonas putida* | Amino acid permease | Transport of amino acids into the cell |  | *Mycobacterium smegmatis* | 1341 | 520569-521912 |
|  | *Vibrio cholerae* | Conserved hypothetical hydrolase-amidase | Hydrolysis of CO-NH_2_ bond with production of NH_3_ | Information storage and processing | *Mycobacterium marinum* | 984 | 3121055-3122041 |
|  |  |  |  |  | *Mycobacterium ulcerans* |  | 3559252-3560238 |
|  | *Legionella pneumophila* | Amidase |  |  | *Mycobacterium marinum* | 1404 | 3367446-3368852 |
|  |  | Pyridine nucleotide-disulfide oxidoreductase | Pyridine nucleotide-disulfide oxidoreductase implicated in oxidative metabolism and disulphide reductase activities | Metabolism | *Mycobacterium smegmatis* | 1041 | 621750-622790 |
|  | *Coxiella burnetii* |  |  |  | *Mycobacterium ulcerans* | 1101 | 4098358-4099458 |
| Eukaryotes | *Dictyostelium discoideum* |  |  |  | *Mycobacterium marinum* | 1101 | 4603844-4604944 |
|  |  |  |  |  | *Mycobacterium abscessus* | 1038 | 673330-674367 |
|  |  |  |  |  | *Mycobacterium intracellulare* | 1047 | 3251943-3252989 |
|  |  |  |  |  | *Mycobacterium avium* | 1011 | 1674723-1675733 |
