# Supplementary material for: The Genealogic Tree of Mycobacteria Reveals a Long-Standing Sympatric Life into Free-Living Protozoa
Source: PLoS One. 2012 Apr 12;7(4):e34754. doi: 10.1371/journal.pone.0034754 (PMC3325273; doi:10.1371/journal.pone.0034754)
Supplement: Table S3 — Description of the nine probably transfered genes. (DOC) [file pone.0034754.s010.doc]

**Table S3. Description of the nine probably transfered genes.**

| **HGT ORFs** | **Bacteria** | **Genome length (Mb)** | **GC% genome** | **Gene length (pb)** | **GC% gene** | **Name of gene** | **Presence of HGT vehicule (Yes or No)** | **Nature of vehicule (position/gene)** |
| --- | --- | --- | --- | --- | --- | --- | --- | --- |
| Pyridine nucleotide disulfide oxidoreductase | *Mycobcaterium abscessus* | 5.07 | 64 | 1038 | 62 | MAB_0670 | N | - |
|  | *Mycobcaterium smegmatis* | 6.98 | 67 | 1041 | 64 | MSMEG_0543 | Y | Transposase (4) |
|  | *Mycobcaterium ulcerans* | 5.63 | 65 | 1101 | 62 | MUL_3683 | Y | Transposase(5) |
|  | *Mycobcaterium marinum* | 6.63 | 65 | 1101 | 63 | MMAR_3739 | N | - |
|  | *Mycobcaterium avium* 104 | 5.47 | 68 | 1011 | 66 | MAV_1699 | Y | Transposase(9) |
|  | *Mycobcaterium intracellulare* | 5.33 | 67 | 1047 | - | MintA_010100015996 | N | - |
| Sulfate transporter | *Mycobacterium bovis* | 4.34 | 65 | 1458 | 62 | Mb1734 | N | - |
|  | *Mycobacterium tuberculosis* H37Rv | 4.41 | 65 | 1458 | 62 | Rv1707 | N | - |
| Betalactamase | *Mycobacterium abscessus* | 5.07 | 64 | 876 | 63 | MAB_2875 | N | - |
| Acetyl-CoA hydrolase | *Mycobacterium marinum* | 6.63 | 65 | 1335 | 61 | MMAR_4160 | N | - |
|  | *Mycobacterium ulcerans* | 5.63 | 65 | 1335 | 61 | MUL_4023 | Y | Transposase(6) |
| AraC family protien, transcriptional regulator | *Mycobacterium smegmatis* | 6.98 | 67 | 849 | 69 | MSMEG_5465 | N | - |
| Hypothetical protien MT3512 | *Mycobacterium tuberculosis H37Rv* | 4.41 | 65 | 702 | 59 | Rv3404c | N | - |
| Amino acid permease | *Mycobacterium smegmatis* | 6.98 | 67 | 1341 | 64 | MSMEG_0446 | N | - |
| Conserved hypothetcial hydorlase-amidase | *Mycobacterium marinum* | 6.63 | 65 | 984 | 64 | MMAR_2570 | N | - |
|  | *Mycobacterium ulcerans* | 5.63 | 65 | 984 | 65 | MUL_3191 | N | - |
| Amidase | *Mycobacterium marinum* | 6.63 | 65 | 1404 | 67 | MMAR_2765 | N | - |
